# Room Temperature Hydrogen Gas Sensing via Reversible Hydrogenation of Electrochemically Deposited Polycarbazole on Interdigitated Pt Transducers

**DOI:** 10.3390/s19051098

**Published:** 2019-03-04

**Authors:** Agnieszka Stolarczyk, Tomasz Jarosz, Marcin Procek

**Affiliations:** 1 Department of Physical Chemistry and Technology of Polymers, Silesian University of Technology, 9 Strzody Street, 44-100 Gliwice, Poland; Agnieszka.Stolarczyk@polsl.pl (A.S.); Tomasz.Jarosz@polsl.pl (T.J.); 2 Department of Inorganic Chemistry, Analytical Chemistry and Electrochemistry, Silesian University of Technology, 6 Krzywoustego Street, 44-100 Gliwice, Poland; 3 Department of Optoelectronics, Silesian University of Technology, 2 Krzywoustego Street, 44-100 Gliwice, Poland

**Keywords:** polycarbazole, electropolymerisation, RT hydrogen gas sensor

## Abstract

In this study, polycarbazole (PCz) is presented as a receptor structure for chemoresistive hydrogen sensors. The fabrication of the proposed sensors via electropolymerisation of PCz on interdigitated Pt electrodes is an inexpensive, cost-efficient, and repeatable method. Preliminary results presented in this work show that PCz-based sensors are sensitive to hydrogen gas in the range of 1–4% in air at room temperature. Notably, responses are both relatively high (from approximately 280% for 1% of H_2_) and rapid (response and recovery times for 1% H_2_ from 5 s and up to 32 s, respectively). Results of PCz structures on Pt and Au electrodes prove that the application of Pt electrodes is crucial for observation of sensing effect. A sensing mechanism based on reversible hydrogenation of PCz is proposed to explain the sensor operating principles.

## 1. Introduction

Polycarbazole (PCz) and its derivatives are well-known, electroactive, and photoluminescent polymers, with applications as active layers in organic photovoltaics [1,2], corrosion protection [3], anion [4], and gas sensors [5,6,7]. PCz shows good thermal and photochemical stability, high triplet energy, and relatively high p-type conductivity [8,9].

Both carbazole (Cz) and PCz have been investigated as potential materials for the chemical storage of hydrogen because, in the presence of a catalyst, both the monomer and polymer can undergo reversible hydrogenation [10,11,12]. Interestingly, despite such reports and the varied use of PCz-based gas sensors, no attempts to apply PCz or its derivatives as hydrogen sensing materials have been reported.

The importance of detecting hydrogen gas stems from its flammability and ability to explode in mixtures with air at a wide range of concentrations (flammability limit 4–75% vol. hydrogen in air; explosive level 18.3–59% vol. hydrogen in air) [13,14]. Accordingly, H_2_ concentration should be monitored in the range of 0–4% or higher for reasons of safety. Simultaneously, numerous branches of technology include processes in which hydrogen gas either evolves or is handled directly, including the fabrication and operation of fuel cells, the manufacture of glass and steel, the refinement of petroleum products, the charge of lead batteries, and the welding of atomic hydrogen/oxyhydrogen. As such, both international and national regulations for possible explosive atmospheres require the monitoring of hydrogen concentrations in the air at any relevant sites, in the event of accidental hydrogen release.

In this paper, we present a possible application of PCz as a receptor material for H_2_ gas sensors operating at room temperature. We prepared PCz films on Pt and Au electrodes using electropolymerisation. Obtained sensors were tested for the reaction to H_2_ in the range of 1–4% in air and nitrogen atmospheres. Preliminary results show that application of Pt electrodes and the presence of oxygen have a crucial impact on the observed hydrogen sensing effect of PCz. These results prove that H_2_ sensing at room temperature using PCz on Pt electrodes is possible. Such an application of PCz has not been reported previously in literature. Additionally, the responses of these sensors are relatively high and fast and, when coupled with their inexpensive and repeatable fabrication method, become very attractive for further investigations and applications.

## 2. Materials and Methods

PCz layers were deposited through electrochemical polymerisation from 20 mM Cz (>98%, Sigma Aldrich, St. Louis, MO, USA) solutions in supporting electrolyte -0.1 M tetrabutylammonium tetrafluoroborate (>99%, Sigma Aldrich, St. Louis, MO, USA) in acetonitrile (>99%, Sigma-Aldrich, St. Louis, MO, USA) on transducers with interdigitated electrodes: Pt (IDE-Pt) or Au (IDE-Au) (Dropsens, Herisau, Switzerland), with dimensions of 5 × 5 μm, according to the procedure presented in our pending patent application [15]. On IDE-Pt, two different thicknesses of PCz films were deposited: ‘thinner’ and ‘thicker’, using 5 and 10 polymerisation cycles, respectively.

Cyclic voltammetry was performed using a standard three-electrode cell, with an IDE-Pt or IDE-Ag working electrode, an Ag pseudo-reference electrode, and a Pt coil counter electrode. Measurements were taken on a Metrohm-Autolab PGSTAT100N (Herisau, Switzerland) potentiostat. Prior to measurement, each investigated sample was purged with inert gas while the same gas was passed through the electrochemical cell during measurement.

IR spectroscopy was carried out on Perkin-Elmer Spectrum Two (Waltham, MA, USA) spectrometer with a UATR (Single Reflection Diamond) module. The morphology of the films was investigated using scanning electron microscopy (SEM), Inspect S50, FEI (Hillsboro, OR, USA).

Obtained sensing structures were tested by measuring their reaction to H_2_ in a concentration range of 1–4% (v/v) in a carrier gas (synthetic air or nitrogen). The measurement stand for gas sensing tests is described in detail elsewhere [16]. The resistance of the sensors was measured using Agilent 34970A in gas flow (constant flow rate = 500 mL/min) at room temperature (RT = 23(±1) °C) and constant gas humidity (RH = 7(±1)%). Measurement cycles consisted of 30 min flow of the carrier gas and 30 min flow of the carrier gas with the indicated and constant concentration of H_2_ (mass flow controllers were used for dosing). The response of the sensor was calculated on the basis of the resistance of the sensor (R_G_) changes in regard to its initial resistance (R_A_) using the following equation:

(1) $$\mathrm{Response}=\frac{R_{G}-R_{A}}{R_{A}}\cdot 100\%$$

Response and recovery times (t_90%resp_ and t_90%rec_, respectively) were calculated as times after which 90% of signal changes was achieved.

## 3. Results and Discussion

### 3.1. Material Identification

**Figure 1:**
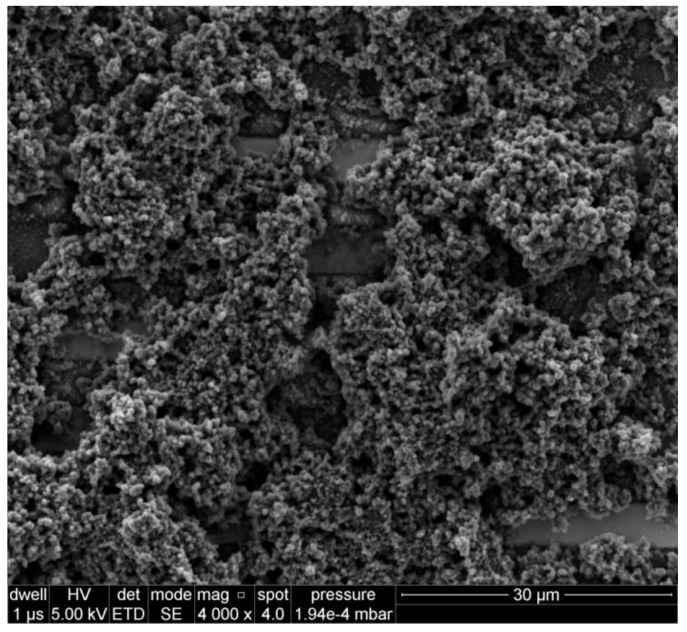
SEM image of PCz deposited on IDE-Pt using 10 polymerisation cycles.

**Figure A1:**
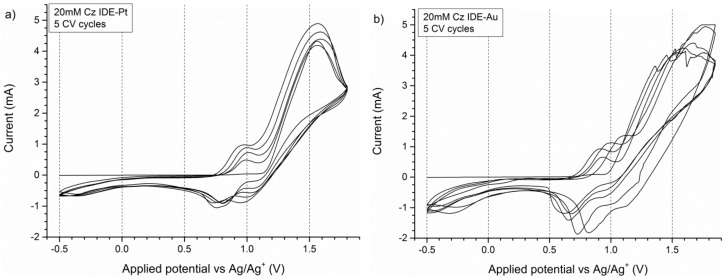
CV curves recorded during polymerisation CZ on transducers with interdigitated electrodes: (**a**) Pt (IDE-Pt); (**b**) Au/Cr (IDE-Au).

**Figure A2:**
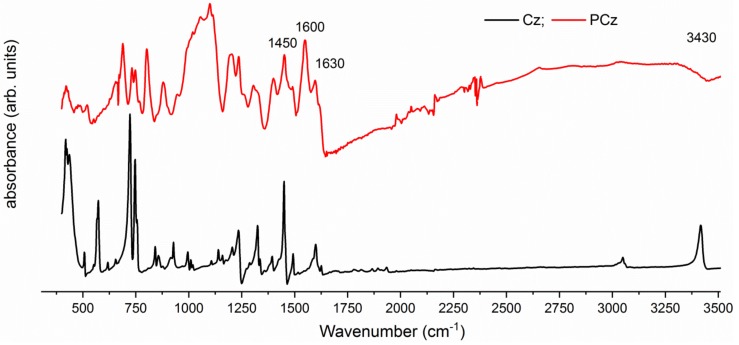
IR-ATR of carbazole (black line) and electrochemical polymerised polycarbazole (red line)

**Figure A3:**
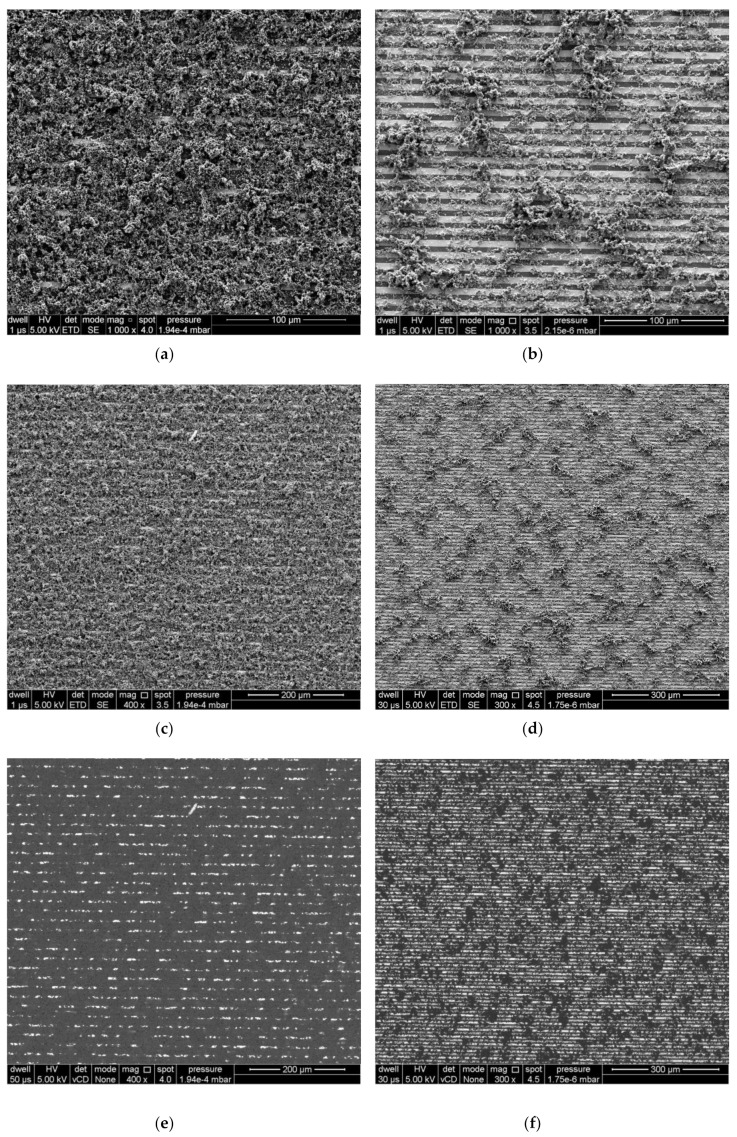

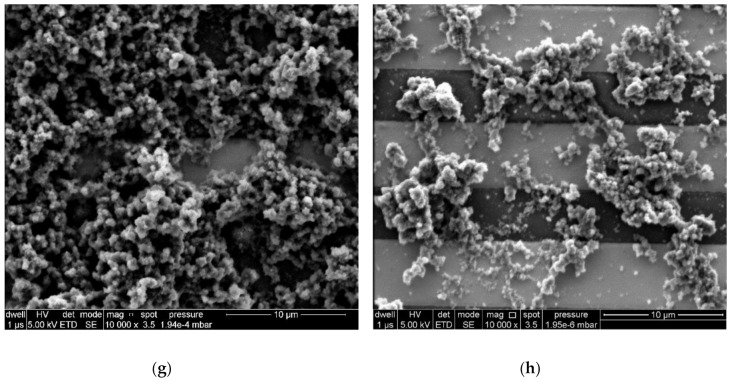
SEM images of PCz electropolymerised on IDE-Pt for: (**a**), (**c**), (**e**), (**g**) ‘Thicker’ film (10 cycles of polymerisation) in different magnifications using Everhart-Thornley Detector ETD (secondary electrons) and low Voltage high Contrast Detector (vCD) (back scattered electrons) detectors (**b**), (**d**), (**f**), (**h**) ‘Thinner’ film (5 cycles of polymerisation in different magnifications using ETD (secondary electrons) and vCD (back scattered electrons) detectors

The cyclic voltammetry curves recorded during polymerisation are included as Figure A1. The shape of the recorded curves is typical for the electrochemical polymerisation of Cz and is in agreement with literature [8]. The structure of PCz was confirmed by IR ATR spectroscopy (Figure A2). IR spectra of the PCz [17] film show an intensive N-H stretching band at 3430 cm^−1^ and a C-H stretching band at 1630 cm^−1^. The bands for C-C and C-N stretching were observed in the range of 1600 to 1450 cm^−1^, and a C-H stretching for the trisubstituted ring was observed in the range of 800 to 750 cm^−1^. The morphology of the films was investigated using SEM. The micrographs of layers deposited on IDE-Pt are presented in Figure 1 and Figure A3, showing porous structures formed by PCz. PCz agglomerates homogenously coat the IDE surface regardless of film thickness, with the ’thicker’ film obscured the IDE-Pt to a greater extent than the ’thinner’ one. The apparent large surface area of the obtained films is desirable for gas sensing applications.

### 3.2. Gas Sensing Properties

**Figure 2:**
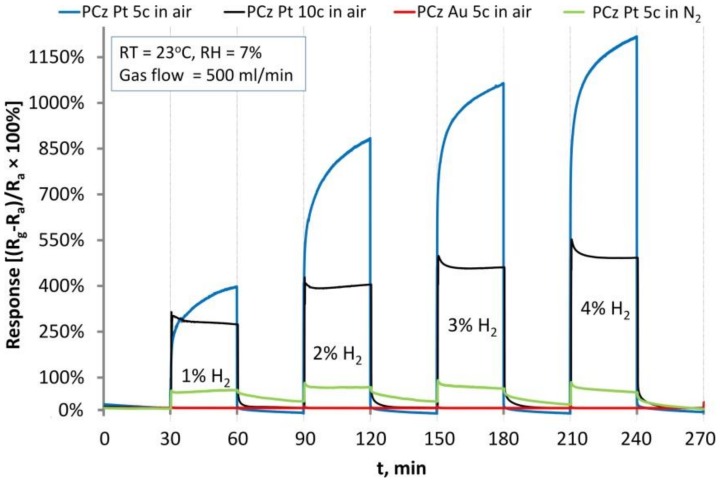
Response of representative (average performance) PCz/IDE-Au and PCz/IDE-Pt sensors to H_2_ gas.

The PCz film deposited on IDE-Au showed no response to hydrogen gas (Figure 2, red line). When PCz was deposited on IDE-Pt, a very strong response (electrical resistance increase) to the presence of hydrogen was observed.

The shape and magnitude of the signal were a function of PCz film thickness (degree of IDE coverage), with ‘thicker’ (black line) and ‘thinner’ (blue line) films giving weaker, rectangular, and stronger, sloped responses, respectively. When nitrogen was used as the carrier gas (green line), the response was lower and recovery of the sensor was very slow, indicating that oxygen played a role in the sensing mechanism.

**Figure A4:**
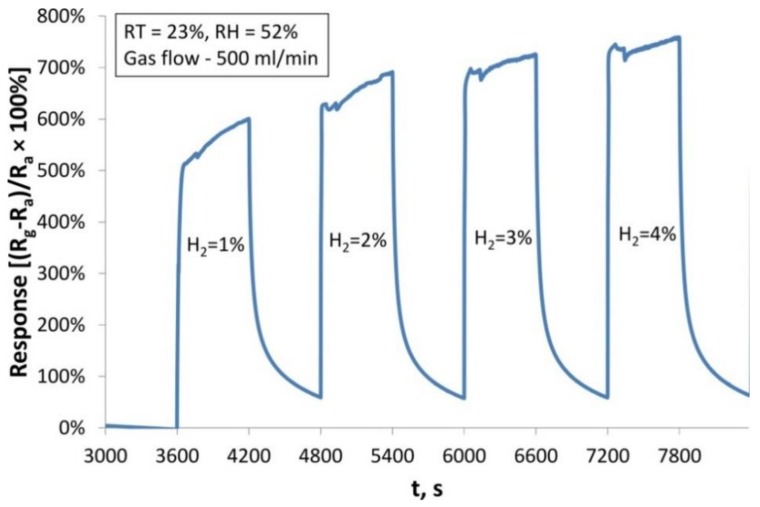
Response of ‘thicker’ PCz/IDE-Pt sensor to H_2_ gas at RH = 52% at RT.

Response, t_resp90%_ and recovery, t_rec90%_ times of the investigated PCz films on IDE-Pts in dry and humid conditions (Figure A4) are collected in Table 1. ‘Thicker’ films responded rapidly to H_2_ and recovered at a decent rate while ‘thinner’ films responded more slowly and recovered more rapidly. We attribute this to the difference in film morphology: the PCz agglomerates (forming potential conductance paths) comprising the ‘thinner’ film were fewer in number and smaller than those comprising the thicker film. Consequently, relative changes of the resistance were higher for the thinner film, but saturation by hydrogen was harder because of easier film penetration by oxygen and hydrogenation/dehydrogenation competition. On the other hand, diffusion of oxygen and expulsion of hydrogen was more difficult in the thicker film. This discrepancy indicates that adjusting the PCz film thickness will be an important part of optimising the performance of the sensors.

The response of the sensors is scalable, with its magnitude being logarithmically proportional to the concentration of H_2_ in the gas mixture being passed through the system.

In terms of the mechanism underlying the operation of the sensor, the increase in resistance upon exposure to H_2_ is expected because PCz is a p-type conducting polymer. However, the lack of any response on IDE-Au cannot be explained solely by the reduction of charge carriers present in the partially doped PCz film. The lack of response on IDE-Au also precludes a sensing mechanism purely on the basis of physical adsorption of H_2_. Because the use of IDE-Pt appears to be crucial in allowing the PCz layer to sense hydrogen, we suspect the specific nature of platinum is the cause, enabling PCz to sense hydrogen. Of the many properties of Pt, its ability to catalyse hydrogenation and dehydrogenation reactions appears to be the most relevant [18]. This choice of property would imply that a reversible chemical reaction takes place in the active layer. One such reaction is likely and has been reported both for Cz and for PCz - reversible hydrogenation [19,20,21].

**Figure 3:**
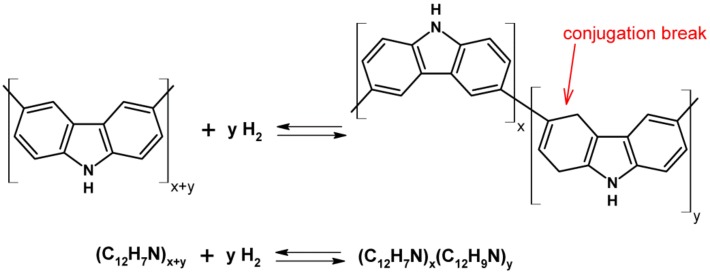
Possible mechanism of sensor operation based on our experimental observations and the properties of carbazole.

In this case, we would observe an increase in the resistance of the active layer, caused not by the reduction of positive charge carriers but by the occurrence of conjugation breaks in the polymer chain, which, in turn, is caused by the hydrogenation of double bonds forming the conjugated bond system (Figure 3). Recovery of the sensor would rely on dehydrogenation of the film. This would be consistent with the shortening of the sensor recovery time seen in the presence of oxygen, as oxygen would be expected to facilitate the dehydrogenation reaction. In Table 2, sensing parameters of different RT H_2_ sensing structures are collated. Different receptor materials, such as metal oxides, carbon nanomaterials, and polymers, were investigated for H_2_ sensing at RT. The production of these structures require relatively expensive, multistep, fabrication processes. Concurrently, these sensor fabrication processes are not necessarily repeatable. In this paper, we show that PCz electropolymerised on IDE-Pt provide comparable or better response values as well as shorter response and recovery times than what has been achieved by other approaches at RT, as seen in Table 1 in comparison with Table 2.

## 4. Conclusions

We have demonstrated the proof of our concept—a working, PCz-based room temperature H_2_ sensor, which has not been previously reported in literature. Our sensor design is competitive with other reported sensors (Table 2) and is easily and repeatedly fabricated from a simple, cost-efficient system. The sensor operates at room temperature, requires no energy draw for heating/cooling, can detect H_2_ concentrations at the 1% level, and is expected to be able to detect even less than 100 ppm concentrations of H_2_.

In terms of sensor optimization, the primary direction is PCz film thickness, as seen by its effect on response and recovery times. Further research into the use of PCz for H_2_ sensing is currently being carried out: sensor selectivity, long-term stability, maximum sensor lifetime, and the effect of environmental conditions (temperature and relative humidity variation) are all under investigation.

## 5. Patents

Pending patent application: Stolarczyk, A.; Jarosz, T.; Procek, M. - Method of obtaining the low temperature chemoresistive hydrogen sensor based on electropolymerised polycarbazole and its derivatives on platinum or palladium transducer, and its application. Polish Pat. pending 2018, P.427906, 1–4.

## Figures and Tables

**Table 1:** Gas sensing properties of representative PCz/IDE-Pt sensors.

Structure	H_2_ Conc.	Resp., %	t_resp90%_, s	t_rec90%_, s
PCz 5c Pt in air (‘thinner’) RH = 7%	1%	398	960	12
	2%	878	740	3
	3%	1056	434	2
	4%	1211	432	2
PCz 10c Pt in air (‘thicker’) RH = 7%	1%	281	5	32
	2%	402	6	30
	3%	460	6	30
	4%	492	7	42
PCz 10c Pt in air (‘thicker’) RH = 52%	1%	593	192	580
	2%	684	10	486
	3%	717	9	474
	4%	748	8	472

**Table 2:** Comparison of the parameters of existing RT H_2_ sensors.

Sensing Structure	H_2_ Concentration (Temperature)	Resp, %	T_resp_, s	T_rec_, s	Ref.
1%Pd-WO_3_	0.5% (RT)	1178	80	10	[22]
CNT on Pt electrodes	0.1% (RT) 0.1% (100°C)	240 359	89 20	39 79	[23]
PMMA/ Pd /graphene	2% (RT)	65	109	331	[24]
Polyaniline/TiO_2_	0.8% (RT)	163	83	130	[25]
